# Supplementary material for: Research on the impact of coupling and coordination of rural consumption power system on the consumption of rural residents in China
Source: PLoS One. 2025 Sep 2;20(9):e0329085. doi: 10.1371/journal.pone.0329085 (PMC12404374; doi:10.1371/journal.pone.0329085)
Supplement: Attachment — Submitted filename: Response to Reviewers.docx [file pone.0329085.s003.docx]

Dear Editor:

During the previous submission of this paper, the reviewer did not make some explicit comments, and amended some text and theoretical mechanisms for the second submission, hoping to be reviewed.

This paper empirically studies the impact of the coupling and coordination of rural consumption power system on the consumption level of rural residents through constructing spatial Durbin model after making theoretical analysis of the function mechanism of the former on the latter.

Few studies have been conducted on farmers’ consumption issues from the perspective of coupling and coordination of rural consumption power systems. This study incorporates consumer subjects, consumer objects, and consumption environment into the consumption power system, analyzes the spatiotemporal evolution of coupling and coordination of rural consumption power systems, and explores the impact of coupling and coordination of consumption power systems on farmers’ consumption.

It would be highly appreciated if you send us any advice regarding this paper.

Yours sincerely,

Dr. Rui Wang
