# Supplementary material for: Research on the impact of coupling and coordination of rural consumption power system on the consumption of rural residents in China
Source: PLoS One. 2025 Sep 2;20(9):e0329085. doi: 10.1371/journal.pone.0329085 (PMC12404374; doi:10.1371/journal.pone.0329085)
Supplement: Attachment — Submitted filename: Minor Revise.docx [file pone.0329085.s004.docx]

I have reviewed this manuscript before. Compared to the previous version, I do see great effort put forth by the author to improve it. Thank you for incorporating my suggestions to the revision of research data and theoretical analysis. However, I still have some concerns that need to be addressed.

1. My major concern is the language used in the revised parts. There are many places that require serious adjustment in order to effectively present ideas. I recommend that professional proofreading is necessary for this paper. The sentences that are confusing and problematic are as follows:

- Page 10, line 14: Change "municipalities" to "municipalities and autonomous regions"
- Page 17: The statistical units or numerical values of urbanization level in Table 3 may not be consistent.

- Page 18, lines 16-17: "According to LM test results, the statistics of LM-Lag, RLM-Lag and RLM-Error tests are all significant at the 1% level, while the statistics of RLM-Error test are significant at the 5% level." In this statement, the level of significance test for RLM-Error is contradictory.
- Page 19: "Based on the panel data of 30 Chinese provinces from 2002 to 2019,......". The year has not been updated.

- Lastly, the recommendation part seems to be solely based on the Chinese context. If the policy recommendations are only for China's national conditions, how about promoting them to other countries?

2. I found no regression results for the robustness test.

3. The author also did not arrange the references in the correct format.
