# Supplementary material for: Research on the impact of coupling and coordination of rural consumption power system on the consumption of rural residents in China
Source: PLoS One. 2025 Sep 2;20(9):e0329085. doi: 10.1371/journal.pone.0329085 (PMC12404374; doi:10.1371/journal.pone.0329085)
Supplement: Attachment — Submitted filename: Response to Reviewers_new.docx [file pone.0329085.s005.docx]

Dear reviewers,

Re: Manuscript ID: **PONE-D-24-26863** &Title: Research on the Impact of Coupling and Coordination of Rural Consumption Power System on the Consumption of Rural Residents in China

Thank you for your comments concerning our manuscript. These comments are all valuable and very helpful for us to revise and improve our paper, which has important guiding significance to us. We have studied comments carefully and made improvements on the paper accordingly, which we hope can meet with your approval. All the revised parts are marked in red in the paper. The main improvements on the paper and the responses to the reviews are as follows:

**Responses to the comments of Reviewer #1:**

Many thanks for your comments. The following are specific responses to each comment.

1. Abstract: The abstract part is suggested to re-describe. It is necessary to emphasize the innovation, importance and outstanding research results of the research, and select several key data to explain when necessary. In addition, it is recommended to emphasize the significance of the study in the end.

**Response:** We revised the abstract by emphasizing the innovation, importance and significance of the research.

2. Introduction: The introduction lacks an introduction to the international context. The introduction does not emphasize the innovativeness of the article. The introduction needs to add the study of the former to prove the feasibility of the study. In addition, the last part of the introduction suggests adding explanations to the rest of the article.

**Response:** We revised the introduction part by adding the analysis of the international context, summarizing the existing research and explaining the structure of the paper.

3. Theoretical Analysis and Research hypothesis: This part needs a lot of literature support, it is suggested that the author increase the literature.

**Response:** On the basis of re-combing the relevant literature, we improved the theoretical analysis and research hypothesis.

4. A comprehensive evaluation of the coupling coordination level of China's rural consumption power system: The specific reasons for the selection of indicators need further explanation.

**Response:** We added the specific reasons for the selection of the evaluation index of the coupling coordination level of China's rural consumption power system.

5. The research results of the article all need to be further explained, not just a description of the results.

**Response:** We added the further explanation of the research results in the conclusion part.

6. Research conclusions and implications: This part is proposed to be changed into conclusions and suggestions. The conclusion and suggestion are divided into two parts.

**Response:** The last part of the manuscript has been divided into two parts: research conclusions and policy suggestions.

7. The article needs to be included in the discussion section.

**Response:** We added robustness test analysis to further discuss the reliability of the research results.

8. The article is also in Chinese. I think the author is not serious and needs to be further improved

**Response:** We revised the manuscript according to the review opinions, and the language expression was carefully improved.

**Responses to the comments of Reviewer #2:**

Special thanks to you for your favorable comments. The following is the specific response.

1.Unlike other related qualitative studies that primarily focus on rural consumption power and the consumption level of rural residents, this manuscript contributes to the literature by constructing and measuring a comprehensive index of the coupling coordination degree of the rural social consumption power system (D) and examining the indirect effects through a spatial spillover model. However, as the research foundation, the literature review and theoretical analysis parts have not been well elaborated and the author should clarify the logical relationship between rural consumption power and rural residents' consumption and discuss the potential insights we can gain from it.

**Response:** We revised and improved the theoretical analysis and research hypothesis, and added the analysis of the logical relationship between rural social consumption power and rural residents' consumption.

2. The indicator selection needs to be carefully considered. In this paper, the proxy indicators of the core explanatory variable indicator system and the control variables are reused, such as INC, EDU, FIN, URB, etc. This approach may lead to correlation and bring estimation bias, making it difficult to accurately assess the impact effect, and the author should consider using different indicators or methods to avoid this problem.

**Response:** In constructing the coupling coordination degree index system of rural social consumption power, 29 evaluation indicators are selected based on the three subsystems of consumption subject, consumption object and consumption environment. Some of these indicators like income level and education level are also important variables that affect the consumption of rural households, and no other suitable indicators can be found to replace them. However, when we use these indicators to calculate the coupling coordination degree of the rural consumption power, we mainly measure the degree of variation of each indicator rather than the absolute value, and all the indicators are dimensionless by standardized methods. Therefore, it will not lead to correlation.

3. In Table 7, the representation of control variables has changed, and variables such as HEA, CPI, and FSA that have not been described and explained in the previous text have appeared. The author should reconfirm the setting of variables to ensure the credibility of the research conclusion.

**Response:** Due to our negligence, there are some errors in the manuscript. We have carefully proofread and improved the full text.

4. Another primary concern lies with the references. Since most of the references are from Chinese literature written in Mandarin, it may be challenging for international readers to refer to them. Additionally, relying mostly on studies conducted in China may limit the generalizability of the findings.

**Response**: The theory of consumption power is mainly from Marx's classical works, while there is little research findings in the area of neoclassical consumption function theory. Therefore, this research failed to cite much literature of western scholars. In addition, as China is a developing country, its unbalanced economic and social development has led to a low level of coupling and coordination of rural consumption power systems to a certain extent, which limits the expansion of rural consumption. The case study about China can not only provide the basis for the policy measures to expand China's rural consumption, but also has certain reference value for other developing countries.

5. Some key references appear to be omitted. I noticed that some studies of well-known authors are mentioned in this paper but not cited, such as Adam Smith, Hermann Haken, and others. Additionally, the citation order of references is disordered and not consistent with the numbers in the article. It is recommended that the author recheck the reference format and citation paradigm.

**Response:** We have improved the quality, quantity and format of the reference

6. We note that the research data is from 2002 to 2019, and based on the data sources in the article, we believe that the author can update the study data to the most recent year.

**Response:** All the data in the manuscript has been updated to 2022.

7. In order to verify the reliability of the research results, the author should adopt various methods to conduct robustness tests, such as replacing the explained variable with survival or development consumption and replacing the core explanatory variable with consumption power lag term.

**Response:** We test the robustness of the model by means of replacing the explained variable with the proportion of enjoyment-type and development-type consumption in the total consumption expenditure. The empirical results are basically consistent with the previous estimation results. Therefore, it can be considered that the empirical results are robust and the research conclusions are reliable.

8. On page 2 and page 22, the phrase "Therefore, neither 'demand-side management' based solely on consumption subjects nor 'supply-side structural reform' based on consumption objects and consumption environments can……in China." appears to overstate the research results of the manuscript, as it does not clearly demonstrate the influence of different subsystems on the consumption level of rural residents.

**Response:** Some of the statements in the manuscript are not accurate enough, and we have modified them according to the comments of the reviewer.
