# Supplementary material for: Research on the impact of coupling and coordination of rural consumption power system on the consumption of rural residents in China
Source: PLoS One. 2025 Sep 2;20(9):e0329085. doi: 10.1371/journal.pone.0329085 (PMC12404374; doi:10.1371/journal.pone.0329085)
Supplement: Attachment — Submitted filename: Response_to_Reviewers_auresp_2.docx [file pone.0329085.s006.docx]

Dear reviewers,

Re: Manuscript ID: **PONE-D-24-26863** & Title: Research on the Impact of Coupling and Coordination of Rural Consumption Power System on the Consumption of Rural Residents in China

Thank you for your comments concerning our manuscript. These comments are all valuable and very helpful for revising and improving our paper, which has important guiding significance to our researches. We have studied comments carefully and made improvements accordingly, which we hope meet with your approval. All the revised parts are marked in red in the paper. The main corrections in the paper and the responses to the reviews are as follows:

1.My major concern is the language used in the revised parts. There are many places that require serious adjustment in order to effectively present ideas. I recommend that professional proofreading is necessary for this paper. The sentences that are confusing and problematic are as follows:

**Response:** The manuscript has undergone professional language refinement, with meticulous revisions made to address suboptimal linguistic formulations specifically highlighted by the reviewers. Additionally, the manuscript supplements the promotion value of this study in the final policy recommendations.

2. I found no regression results for the robustness test.

**Response:** We test the robustness of the model by replacing the explained variables with the proportion of enjoyment and development consumption. The results of the robustness test are detailed on page 24.

3. The author also did not arrange the references in the correct format.

**Response:** We have laid out all the references in APA format.
