# Supplementary material for: Research on the impact of coupling and coordination of rural consumption power system on the consumption of rural residents in China
Source: PLoS One. 2025 Sep 2;20(9):e0329085. doi: 10.1371/journal.pone.0329085 (PMC12404374; doi:10.1371/journal.pone.0329085)
Supplement: Attachment — Submitted filename: Response to Reviewer.docx [file pone.0329085.s008.docx]

Dear reviewers,

Re: Manuscript ID: **PONE-D-24-26863** &Title: Research on the Impact of Coupling and Coordination of Rural Consumption Power System on the Consumption of Rural Residents in China

Thank you for your comments concerning our manuscript. These comments are all valuable and very helpful for us to revise and improve our paper, which has important guiding significance to us. We have studied comments carefully and made improvements on the paper accordingly, which we hope can meet with your approval. All the revised parts are marked in red in the paper. The main improvements on the paper and the responses to the reviews are as follows:

**Q:** In order to verify the reliability of the research results, the author should adopt various methods to conduct robustness tests, such as replacing the explained variable with survival or development consumption and replacing the core explanatory variable with consumption power lag term.

**Response:** We test the robustness of the model by means of replacing the explained variable with the proportion of enjoyment-type and development-type consumption in the total consumption expenditure. The empirical results are basically consistent with the previous estimation results. Therefore, it can be considered that the empirical results are robust and the research conclusions are reliable.

To ensure the robustness of the empirical results, this paper takes the consumption structure of rural residents as the substitution variable of the explained variable and applies the spatial Durbin model to test the impact of the coupling and coordination of rural social consumption power on the consumption of rural residents. The consumption structure of rural residents is measured by the proportions of development-type consumption and enjoyment-type consumption in residents’ living consumption expenditure. The results demonstrate that the total effect coefficient of the coupling and coordination of rural social consumption system on the consumption structure of rural residents is 0.238. The hypothesis test is verified at the significance level of 1%, which indicates that the higher the coupling and coordination level of rural social consumption system in one province is, the more optimized the consumption structure of rural residents is. The spatial lag coefficient of the coupling and coordination level of the rural consumption power system is 0.394 and the null hypothesis is rejected at the significance level of 1%, which indicates that the coupling and coordination development level of the rural consumption power system has significant spatial effect. This result shows that the coupling and coordination development level of the rural consumption power system in one province can play a significant role in promoting the optimization of rural residents’ consumption structure of its neighbouring provinces. This research further uses the partial differential method to decompose the impact of the coupling coordination level of the rural consumption power system on the consumption structure of rural residents into direct effect , indirect effect and total effect with values of 0.209, 0.129 and 0.338 respectively, which are significant at the 1% level. This result demonstrates that the coupling coordination level of rural consumption power system not only positively impacts the consumption structure of rural residents in a certain province but also induces significant spatial spillover effect. This conclusion is basically in line with the previous estimation results. It can be considered that the empirical results of this research are robust and the conclusions are reliable.
